# Pre-existing statin use and recurrence in relation to hematoma architecture of chronic subdural hematomas - A propensity score-matched analysis

**DOI:** 10.1016/j.bas.2026.106048

**Published:** 2026-04-11

**Authors:** Hussam Hamou, Hani Ridwan, Anna Mausberg, Kimberley Fay-Rodrian, Hans Clusmann, Anke Hoellig, Michael Veldeman

**Affiliations:** a Department of Neurosurgery, RWTH Aachen University Hospital, Aachen, Germany; b Department of Diagnostic and Interventional Neuroradiology, RWTH Aachen University, Aachen, Germany

**Keywords:** Chronic subdural hematoma, Statins, Recurrence, Hematoma architecture, Propensity score matching

## Abstract

**Introduction:**

Chronic subdural hematoma (cSDH) affects increasing patient numbers annually, with postoperative recurrence rates of 5-33%. While atorvastatin shows efficacy as primary conservative treatment, the prognostic value of pre-existing statin use in surgical candidates remains unclear.

**Research question:**

This study examined associations between pre-existing statin therapy, hematoma architecture on computed tomography, and postoperative recurrence risk.

**Material and methods:**

This retrospective cohort study analyzed 564 consecutive patients surgically treated for cSDH at a tertiary center (2015-2023). Pre-existing statin use was documented and hematoma architecture classified into homogeneous, organized, sedimented, and subacute subtypes. The primary outcome was recurrence requiring reoperation. Multivariable logistic regression with hierarchical adjustment evaluated associations between statin use and recurrence, adjusting for demographics, comorbidities, medications, and hematoma characteristics. Interaction analyses tested whether statin effects varied by architecture. Propensity score matching (1:1, n = 252) reduced confounding.

**Results:**

Statin use was documented in 150 patients (26.6%). Recurrence occurred in 170 patients (30.1%): 32.9% in non-users versus 22.7% in users. Unadjusted analysis suggested protection (OR 0.599, p = 0.021), but this attenuated after adjusting for cardiovascular comorbidities (OR 0.649, p = 0.076) and became non-significant after full adjustment (OR 0.664, p = 0.109). Propensity matching corroborated findings (OR 0.736, p = 0.269). Hematoma architecture was the dominant predictor: organized hematomas showed 70% lower recurrence risk (OR 0.303, p < 0.001). No differential statin effects across subtypes were observed (p = 0.668).

**Discussion and conclusion:**

Pre-existing statin therapy shows no independent association with postoperative recurrence after adjusting for confounders and hematoma architecture. Prospective investigation of post-surgical statin initiation is warranted.

**Trial registration:**

The study was registered with the German Clinical Trials Register (DRKS00025280).

## Highlights

• Pre-existing statins don't reduce recurrence after cSDH surgery independently
• Hematoma architecture is the strongest predictor of postoperative recurrence
• Organized hematomas show 70% lower recurrence risk versus homogeneous types
• Propensity score matching confirms no independent statin-recurrence association
• Post-surgical statin initiation for recurrence prevention needs investigation

## 1 Introduction

Chronic subdural hematoma (cSDH) represents one of the most common intracranial conditions requiring neurosurgical intervention, with an estimated annual incidence of 1.72 to 20.6 per 100,000 individuals (Yang and Huang, 2017). Driven by population aging and the increasing prescription of antithrombotic medications, the global burden of cSDH continues to escalate, contributing to rising healthcare costs (Adhiyaman et al., 2017; Dulleston et al., 2025). cSDH pathophysiology typically involves a minor head trauma, leading to rupture of bridging veins or disruption of the dura arachnoid interface (Edlmann et al., 2017). Many of the resulting small acute subdural collections resolve spontaneously, however, factors such as brain atrophy, antithrombotic therapy, epilepsy, and alcohol abuse can drive hematoma liquefaction and progressive enlargement (Sim et al., 2012). Injury to dural border cells appears central to fibroproliferation and formation of vascularized neomembranes that promote recurrent bleeding (Huang et al., 2024; Nouri et al., 2021). Burr hole evacuation with drainage remains the standard treatment for symptomatic cSDH. Postoperative recurrence requiring reoperation occurs in approximately 5 to 33 percent of cases (Kolias et al., 2014). Factors associated with recurrence include larger hematoma volume, antithrombotic medication use, seizures, postoperative incomplete brain re-expansion, and less organized hematoma architecture on computed tomography (CT) scanning. Hematoma organization and membrane formation are commonly understood as stages of a spontaneous repair process, with organized subtypes showing reduced recurrence risk (Hamou et al., 2022a; Nakaguchi et al., 2001). Treatment is currently undergoing a shift as middle meningeal artery embolization in selected patients is demonstrating positive results as an adjunct to surgery or in patients not eligible for surgery (Kan et al., 2024). Experimental data provide a mechanistic basis for statin therapy in cSDH (Wang et al., 2021a; Yang et al., 2022; Li et al., 2014). In animal models, atorvastatin accelerates hematoma absorption, improves neurological recovery, reduces inflammation, and promotes neovascularization. Further work shows that its effects involve macrophage polarization toward reparative M2 phenotypes through CSF-1R signaling (Yang et al., 2022). The ATOCH trial demonstrated that atorvastatin 20 mg daily for eight weeks reduced hematoma volume more than placebo in conservatively managed patients, and improved neurological outcomes (Jiang et al., 2018). Subsequent studies, including evidence of synergy with low-dose dexamethasone and registry data showing improved functional outcomes and lower mortality, further support statins’ therapeutic potential (Wang et al., 2021b). These studies focused on statin therapy initiated after diagnosis. In contrast, the impact of pre-existing statin use in patients undergoing surgical treatment for cSDH remains unclear, and whether statins are associated with differences in hematoma membrane internal architecture has not been systematically investigated.

### 1.1 Objectives

The objective of this study was to evaluate prognostic relevance of chronic background use of statins in a cohort of patients with cSDH who were surgically treated. In addition, we wish to assess the associations between pre-existing statin therapy and hematoma internal architecture on CT.

## 2 Methods

### 2.1 Study design and participants

This retrospective cohort analysis included all consecutive patients treated surgically for cSDH at RWTH Aachen University Hospital from January 2015 through December 2023. The investigation builds on previously published institutional cohorts (Hamou et al., 2022a, 2022b; Veldeman et al., 2024), with the present study specifically focusing on associations between pre-existing statin therapy, hematoma architecture, and postoperative recurrence risk. Our local ethics committee granted approval (EK20/399 & EK 25/331). Given the retrospective study design, individual informed consent requirements were waived. The study was registered with the German Clinical Trials Register (DRKS00025280).

Patients were eligible if they had a computed tomography–confirmed hematoma treated surgically by burr hole craniotomy or twist drill craniostomy. Exclusion criteria included the absence of preoperative CT imaging; intracranial hypotension contributing to hematoma formation; prior cranial surgery or procedures causally related to hematoma development; or non-iatrogenic bleeding disorders, including hepatogenic or inherited coagulopathies.

### 2.2 Data collection

Clinical and imaging data were systematically extracted from electronic health records. Collected patient-level variables included demographic information, history of head trauma, clinical symptoms and neurological examination finding, admission Glasgow Coma Scale (GCS), and pre-existing comorbidities. Pre-admission medication profiles were documented with particular attention to antiplatelet therapy (monotherapy and dual antiplatelet therapy), oral anticoagulation (vitamin K antagonists and direct oral anticoagulants), and statin therapy. Admission laboratory parameters included activated partial thromboplastin time, international normalized ratio, and platelet count.

### 2.3 Radiological evaluation

Preoperative CT imaging was systematically analyzed for multiple radiological parameters. Assessed variables included hematoma laterality (unilateral *versus* bilateral), maximum transverse diameter, craniocaudal extent, and total hematoma volume (Brainlab, Munich, Germany).

Internal hematoma architecture was classified according to our previously validated extended typology comprising eight distinct patterns: bridging, subacute, laminar, trabecular, hyperdense, isodense, hypodense, and sedimented (Hamou et al., 2022a). Two independent raters (HH and MV), blinded to clinical outcomes, performed the classification. For analytical purposes, the eight-category classification was consolidated into four groups: homogeneous (encompassing hypodense, isodense, and hyperdense appearances), organized (comprising laminar, bridging, and trabecular patterns), sedimented, and subacute.

### 2.4 Surgical protocol and postoperative management

Surgical evacuation was indicated for patients presenting with neurological deficits (motor weakness, gait disturbance, speech impairment, or seizures) or for asymptomatic patients with radiographic evidence of mass effect (including midline displacement, ventricular compression, or sulcal effacement). Isolated headache without objective neurological deficits or mass effect did not constitute an operative indication.

The standard surgical approach consisted of burr hole craniotomy with irrigation and placement of one or two passive subdural silicone drains (12-French) under general anesthesia or monitored sedation. Single drain placement was use when intraoperative brain re-expansion limited the residual subdural space. Twist drill craniostomy under local anesthesia without irrigation was reserved for homogeneous hematomas in patients with substantial medical comorbidity. No patients underwent middle meningeal artery embolization.

Routine postoperative imaging during the index hospitalization was obtained for patients treated with twist drill craniotomy and selectively for burr hole patients who developed new neurological symptoms, had persistent preoperative deficits, or in whom inadequate evacuation was clinically suspected. Significant residual hematoma with persistent mass effect prompted revision surgery during the same hospitalization admission.

Discharge criteria included complete symptom resolution or postoperative imaging demonstrating resolution of mass effect resolution. After discharge, follow-up CT imaging was performed 21 to 28 days postoperatively and repeated at 4-week intervals until radiological resolution.

### 2.5 Outcome definition

The primary outcome was postoperative recurrence requiring surgical reintervention. Recurrence was defined as volumetric enlargement of a residual or newly accumulated subdural collection associated with radiographic mass effect and/or new onset or worsening neurological symptoms necessitating repeat surgical evacuation. Reintervention consisted of burr hole craniotomy with subdural drain placement.

### 2.6 Data analysis

All statistical analyses were performed using R version 4.5.2 (R Foundation for Statistical Computing, Vienna, Austria) within RStudio (version 2025.09.2 + 418, Posit Software, PBC, Boston, MA). Primary packages included the *tidyverse* along with *MatchIt* for propensity score matching (PSM) and *cobalt* for covariate balance assessment.

Continuous variables were summarized as means with standard deviations (SD) for normally distributed data or medians with interquartile ranges (IQR, Q1–Q3) for non-normally distributed data. Distribution normality was assessed by visual inspection of histograms and, when appropriate, using the Shapiro-Wilk test. Categorical variables were reported as frequencies and percentages.

Baseline patient characteristics, imaging features, and surgical variables were compared between patients with and without recurrence using appropriate bivariate tests. Independent-samples t tests were applied for normally distributed continuous variables, Mann-Whitney U tests for non-normally distributed continuous variables, and Pearson's chi-square tests (or Fisher's exact tests when expected cell counts were **<** 5) for categorical variables.

The independent association between statin use and postoperative recurrence was evaluated using multivariable binary logistic regression, with recurrence requiring reoperation as the dependent variable. An iterative hierarchical modeling strategy was employed, sequentially incorporating pre-specified covariate blocks to assess potential confounding. Models were built sequentially, adding theoretically relevant variable groups at each step. Nested models were compared using likelihood ratio tests to determine whether additional covariate groups significantly improved model fit. Akaike Information Criterion (AIC) was used to compare non-nested models, with lower values indicating superior model fit. These metrics guided selection of the most parsimonious final model. Multicollinearity was assessed using variance inflation factors (VIF), with values exceeding 5.0 indicating problematic collinearity requiring remediation. Results were reported as odds ratios (OR) with 95% confidence intervals (CI). The homogeneous hematoma type served as the reference category for the extended hematoma architecture variable. Statistical significance was defined as a two-sided p-value <0.05.

### 2.7 Propensity score matching

To address potential confounding by indication and minimize systematic differences between patients receiving and not receiving statins, propensity score matching was performed (PSM) as a sensitivity analysis. The propensity score, representing the conditional probability of statin exposure given observed covariates, was estimated using a logistic regression model.

Variables incorporated in the propensity score model included: patient demographics (age, sex), cardiovascular comorbidities (hypertension, coronary artery disease, cardiac arrhythmias, diabetes mellitus, prior myocardial infarction, prior stroke), other medication use (antiplatelet therapy, oral anticoagulation, angiotensin-converting enzyme inhibitors, angiotensin receptor blockers), hematoma imaging characteristics (volume, laterality, extended hematoma type, midline shift), and clinical presentation features (Glasgow Coma Scale score, history of trauma).

One-to-one nearest neighbor matching without replacement was performed using a caliper width of 0.2 standard deviations of the logit of the propensity score to ensure adequate match quality while maximizing sample retention. Balance before and after matching was assessed using standardized mean differences (SMD), with absolute SMD values less than 0.1 indicating adequate balance. Distributional balance of propensity scores before and after matching was visualized using density plots.

Following PSM, the association between statin use and recurrence was re-evaluated using logistic regression in the matched cohort, with weights applied to account for the matching structure. Results from the matched analysis were compared to the primary unmatched multivariable model to assess robustness of findings to residual confounding.

All statistical tests were two-sided, and p-values <0.05 were considered statistically significant unless otherwise specified. Forest plots were generated to visualize odds ratios and confidence intervals across different analytical approaches.

## 3 Results

### 3.1 Patient cohort and baseline characteristics

**Fig. 1:**
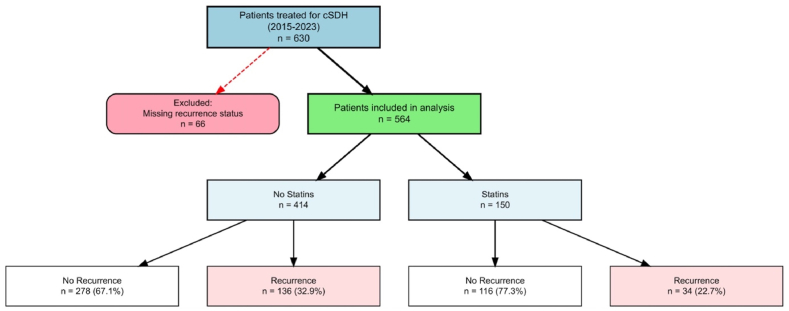
**Patient Flow Diagram** Flow chart depicting patient selection and outcomes in the study cohort. The final analytical cohort of 564 patients was stratified by pre-existing statin use at the time of presentation: patients (73.4%) were not receiving statins and 150 patients (26.6%) were receiving statin therapy. Recurrence rates requiring surgical reintervention were 32.9% (136/414) in the non-statin group and 22.7% (34/150) in the statin group.

Between January 2015 and December 2023, a total of 630 patients underwent surgical treatment for chronic subdural hematoma at our institution. Of these, 66 patients (10.5%) were excluded due to missing documentation of recurrence status, resulting in a final analytical cohort of 564 patients (Fig. 1). The study population had a mean age of 74.6 ± 11.8 years and comprised 369 males (65.4%) and 195 females (34.6%). Overall, 170 patients (30.1%) experienced postoperative recurrence requiring surgical reintervention. Pre-existing statin therapy at the time of cSDH presentation was documented in 150 patients (26.6%), while 414 patients (73.4%) were not receiving statins. Among the 150 patients on statin therapy, the most commonly prescribed agents were simvastatin (n = 89, 59.53%), atorvastatin (n = 52, 34.7%), rosuvastatin (n = 4, 2.7%), and pravastatin (n = 2, 1.3%), missing (n = 3, 2.0%).

Baseline patient demographics, clinical characteristics, imaging features, and comorbidity profiles stratified by statin use are detailed in Table 1. Patients receiving statins were significantly older (mean age 79.5 (73.2 to 84.0) years vs 76.5 (67.0 to 83.0) (p < 0.001) and demonstrated higher prevalence of cardiovascular comorbidities, including hypertension (81.3% vs 55.0%, p < 0.001), and diabetes mellitus (30.7% vs 13.1%, p < 0.001). Consistent with their cardiovascular disease burden, statin users were more frequently treated with antiplatelet agents (62.0% vs 22.0%, p < 0.001) and oral anticoagulation (18.8% vs 15.0%, p = 0.343). Angiotensin-converting enzyme inhibitors or angiotensin receptor blockers were also more prevalent among statin users (63.3% vs 24.2%, p < 0.001). Despite these clinical differences, no significant disparities were observed between groups regarding hematoma imaging characteristics, including laterality, preoperative volume (median 138.0 mL vs 129.0 mL, p = 0.756), midline shift (yes: 72.2% vs 73.0%, p = 0.952), or extended hematoma type classification (p = 0.658).

**Table 1:** **Baseline Characteristics Stratified by Statin Use** Data are presented as mean ± standard deviation for normally distributed continuous variables, median (interquartile range) for non-normally distributed continuous variables, and number (percentage) for categorical variables. Extended hematoma type classification: homogeneous (hypodense, isodense, hyperdense), organized (laminar, bridging, trabecular), sedimented, and subacute.

Variable	No statins (n = 414)	Statins (n = 150)	p-value
**DEMOGRAPHICS**			
Age (years)	76.5 (67.0 to 83.0)	79.5 (73.2 to 84.0)	0.001
Sex (female)	152 (36.7%)	43 (28.7%)	0.094
**COMORBIDITY**			
Hypertension	227 (55.0%)	122 (81.3%)	<0.001
Diabetes	54 (13.1%)	46 (30.7%)	<0.001
Antiplatelets	91 (22.0%)	93 (62.0%)	<0.001
Oral anticoagulants	62 (15.0%)	28 (18.8%)	0.343
Pre-op aPTT (sec.)	28.9 (26.8 to 31.0)	28.8 (26.9 to 31.1)	0.954
Pre-op INR	1.0 (1.0 to 1.1)	1.1 (1.0 to 1.1)	0.083
Pre-op thrombocytes (/nL)	236.5 (191.0 to 292.8)	229.0 (187.0 to 279.5)	0.207
**CLINICAL PRESENTATION**			
Recall of trauma	298 (72.0%)	112 (74.7%)	0.599
GCS on admission	15.0 (14.0 to 15.0)	15.0 (14.0 to 15.0)	0.451
Seizure before treatment	36 (8.7%)	14 (9.3%)	0.959
Paresis	196 (47.8%)	76 (51.0%)	0.566
Reduced consciousness	114 (29.2%)	36 (24.7%)	0.355
Phatic disorder	95 (23.2%)	37 (24.8%)	0.767
**HEMATOMA CHARACTERISTICS**			
Pre-op volume (mL)	129.0 (90.5 to 180.0)	138.0 (99.5 to 182.0)	0.756
Max. width (mm)	22.0 (17.0 to 26.0)	21.0 (18.0 to 26.0)	0.895
Bilateral	123 (29.7%)	28 (18.7%)	0.012
Midline shift	294 (73.0%)	104 (72.2%)	0.952
Extended type			
homogeneous	171 (41.5%)	58 (40.0%)	0.658
organized	165 (40.0%)	57 (39.3%)	
sedimented	26 (6.3%)	7 (4.8%)	
subacute	50 (12.1%)	23 (15.9%)	
**OUTCOME**			
Post-op ICU stay	262 (63.3%)	78 (52.0%)	0.004
Recurrence	136 (32.9%)	34 (22.7%)	0.026
Dichotomized GOS			
Favorable outcome (GOS 1-2)	349 (84.5%)	123 (83.7%)	0.916
Unfavorable outcome (GOS 3-5)	64 (15.5%)	24 (16.3%)	

ACE, angiotensin-converting enzyme; ARB, angiotensin receptor blocker; ASA, American Society of Anesthesiologists physical status classification; GCS, Glasgow Coma Scale; INR, international normalized ratio; IQR, interquartile range; SD, standard deviation.

### 3.2 Multivariable logistic regression analysis

The association between pre-existing statin use and recurrence was evaluated through iterative multivariable logistic regression modeling (Table 2). In the unadjusted analysis, statin use was associated with significantly reduced odds of recurrence (OR 0.599, 95% CI 0.384–0.916, p = 0.021). This protective association persisted after adjustment for demographic factors including age and sex (Model 2: OR 0.569, 95% CI 0.362–0.877, p = 0.012) (see Table 3).

**Table 2:** **Multivariable Logistic Regression Models: Association Between Pre-existing Statin Use and Postoperative Recurrence** Odds ratios (95% confidence intervals) for recurrence associated with statin use across iterative modeling approaches. Demographics adjusted: age and sex. Comorbidities adjusted: demographics plus hypertension, diabetes, antiplatelet therapy, and oral anticoagulation. Fully adjusted (clinical): comorbidities plus hematoma laterality, preoperative volume, and midline shift. Fully adjusted + hematoma type: clinical model plus extended hematoma type classification (homogeneous, organized, sedimented, subacute). PSM: propensity score-matched analyses (1:1 matching, n = 126 pairs). The crude protective association observed in unadjusted analysis attenuated progressively with adjustment for confounders, becoming non-significant after accounting for cardiovascular comorbidities. Propensity score matching corroborated null findings, confirming no independent association between pre-existing statin use and recurrence.

Model	Dataset	Result	p-value
Unadjusted	Full (n = 564)	0.6 (0.38-0.92)	0.021
Demographics adjusted	Full (n = 564)	0.57 (0.36-0.88)	0.012
Comorbidities adjusted	Full (n = 564)	0.65 (0.4-1.04)	0.076
Fully adjusted (clinical)	Full (n = 564)	0.66 (0.4-1.09)	0.109
Fully adjusted + hematoma type	Full (n = 564)	0.65 (0.38-1.09)	0.105
PSM - Unadjusted	Matched (n = 252)	0.74 (0.42-1.27)	0.269
PSM - With hematoma type	Matched (n = 252)	0.71 (0.4-1.26)	0.25

**Table 3:** **Stratified Analysis of Statin Association with Recurrence by Hematoma Architecture** Recurrence rates and adjusted odds ratios stratified by extended hematoma type classification in the full cohort and propensity score-matched cohort. Adjusted odds ratios control for age, sex, hypertension, diabetes, antiplatelet therapy, and preoperative hematoma volume. Despite numerically lower recurrence rates among statin users in most strata, no statistically significant associations were observed except for subacute hematomas in the matched cohort, which approached significance (OR 0.09, p = 0.052) but was based on only 5 total events. Sedimented and subacute subgroups demonstrated wide confidence intervals reflecting limited sample sizes. CI, confidence interval; OR, odds ratio.

Hematoma Type	Dataset	No Statins (n)	No Statins Recurrence (%)	Statins (n)	Statins Recurrence (%)	Adjusted OR	95% CI	p-value
Homogeneous	Full cohort	171	46.2	58	34.5	0.77	0.37-1.59	0.48
Organized	Full cohort	165	20.6	57	15.8	0.72	0.29-1.68	0.467
Sedimented	Full cohort	26	50	7	42.9	0.39	0.03-3.36	0.41
Subacute	Full cohort	50	20	23	4.3	0.2	0.01-2.42	0.241
Homogeneous	Matched cohort	45	44.4	50	38	0.77	0.34-1.74	0.524
Organized	Matched cohort	59	18.6	48	18.8	1.01	0.37-2.68	0.989
Sedimented	Matched cohort	12	50	7	42.9	0.75	0.11-4.94	0.764
Subacute	Matched cohort	10	40	17	5.9	0.09	0.00-0.79	0.052

However, when cardiovascular comorbidities and concomitant medications were incorporated (Model 3), the association attenuated and lost statistical significance (OR 0.649, 95% CI 0.399–1.04, p = 0.076). Further adjustment for hematoma imaging characteristics including laterality, preoperative volume, and midline shift in the fully adjusted clinical model (Model 4) yielded similar results (OR 0.664, 95% CI 0.399–1.09, p = 0.109). Notably, preoperative hematoma volume emerged as a significant independent predictor of recurrence in this model (OR 1.01 per mL, 95% CI 1.00–1.01, p < 0.001).

Given the established prognostic importance of hematoma architecture (Hamou et al., 2022a; Nakaguchi et al., 2001), we evaluated whether the association between statins and recurrence varied across extended hematoma types. Model 5 incorporated extended hematoma type classification alongside statins and clinical confounders. Hematoma architecture demonstrated strong independent associations with recurrence: organized hematomas showed markedly reduced recurrence risk compared to homogeneous types (OR 0.303, 95% CI 0.192–0.472, p < 0.001), as did subacute hematomas (OR 0.214, 95% CI 0.094–0.440, p < 0.001), while sedimented hematomas showed no significant difference (OR 1.17, 95% CI 0.537–2.54, p = 0.691). In this model accounting for hematoma architecture, statin use remained non-significant (OR 0.652, 95% CI 0.384–1.09, p = 0.105).

To formally test whether statin effects differed by hematoma type, we constructed an interaction model (Model 6) including multiplicative interaction terms between statin use and extended hematoma type. The likelihood ratio test comparing Models 5 and 6 showed no evidence of significant interaction (χ^2^ = 1.56, df = 3, p = 0.668). Additionally, the interaction model demonstrated inferior fit by Akaike Information Criterion (AIC 611.2 vs 606.8), favoring the simpler main effects model.

### 3.3 Stratified analysis by hematoma architecture

**Fig. 2:**
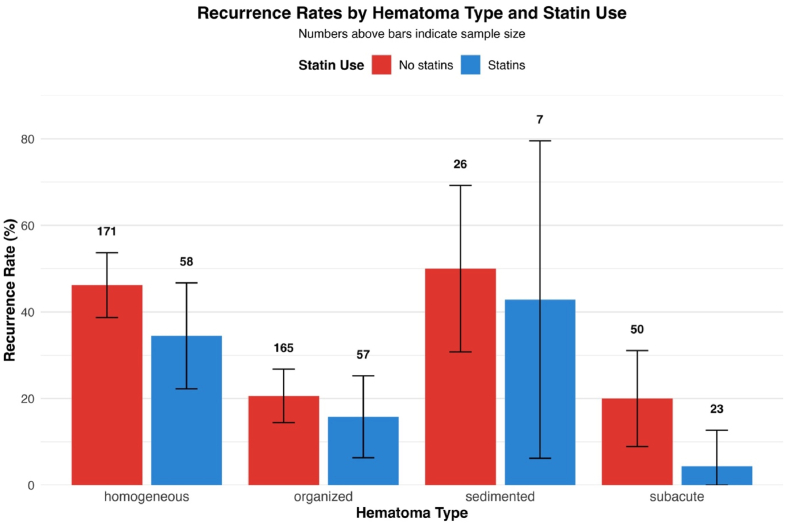
**Recurrence Rates Stratified by Hematoma Architecture and Statin Use** Recurrence rates requiring surgical reintervention stratified by extended hematoma type classification and pre-existing statin therapy. Error bars represent 95% confidence intervals calculated using standard error. Numbers above bars indicate sample size for each subgroup.

Despite the absence of statistical interaction, we performed stratified analyses to examine statin associations within each hematoma architectural subtype (Fig. 2). Baseline recurrence rates varied substantially by hematoma type, with homogeneous hematomas demonstrating the highest recurrence (46.2% without statins, 34.5% with statins), followed by sedimented (50.0% vs 42.9%), organized (20.6% vs 15.8%), and subacute hematomas (20.0% vs 4.3%).

Among homogeneous hematomas (n = 229), the crude odds ratio for statin use was 0.61 (95% CI 0.33–1.13, p = 0.121), which attenuated to 0.77 (95% CI 0.37–1.59, p = 0.480) after adjustment for age, sex, comorbidities, and preoperative volume. For organized hematomas (n = 222), statin use showed no association in either unadjusted (OR 0.72, 95% CI 0.31–1.56, p = 0.429) or adjusted (OR 0.72, 95% CI 0.29–1.68, p = 0.467) analyses.

### 3.4 Propensity score matching sensitivity analysis

To address potential confounding by indication and systematic differences between statin users and non-users, we performed propensity score matching as a sensitivity analysis. After excluding 30 patients (5.3%) with missing data in matching variables, 534 patients remained eligible for matching (392 non-statin users, 142 statin users). Baseline assessment revealed substantial imbalance between groups, with SMD exceeding 0.1 for age (SMD 0.432), sex (SMD 0.157), hypertension (SMD 0.581), diabetes (SMD 0.413), antiplatelet use (SMD 0.885), and laterality (SMD 0.243), indicating meaningful baseline differences requiring adjustment.

One-to-one nearest neighbor propensity score matching with a caliper of 0.2 SDs successfully matched 126 statin users with 126 non-statin controls, yielding a final matched cohort of 252 patients (47.2% of eligible patients). Sixteen statin users (11.3%) remained unmatched due to lack of sufficiently similar controls. Post-matching balance assessment demonstrated excellent covariate balance, with all SMD values below 0.1 except preoperative hematoma volume (SMD 0.120), which remained at an acceptable level.

In the matched cohort, recurrence rates were 32.5% (41/126) among non-statin users and 26.2% (33/126) among statin users. McNemar's test for paired data showed no significant difference between matched pairs (χ^2^ = 1.02, p = 0.312). Logistic regression in the matched cohort yielded an OR of 0.736 (95% CI 0.425–1.27, p = 0.269), consistent with the non-significant finding in the primary unmatched analysis but with wider confidence intervals reflecting the reduced sample size.

When incorporating hematoma architecture into the matched analysis, organized hematomas maintained their strong protective association (OR 0.320, 95% CI 0.166–0.600, p < 0.001), as did subacute hematomas (OR 0.336, 95% CI 0.105–0.905, p = 0.043). Statin use remained non-significant after adjusting for hematoma type (OR 0.714, 95% CI 0.400–1.26, p = 0.250).

**Fig. 3:**
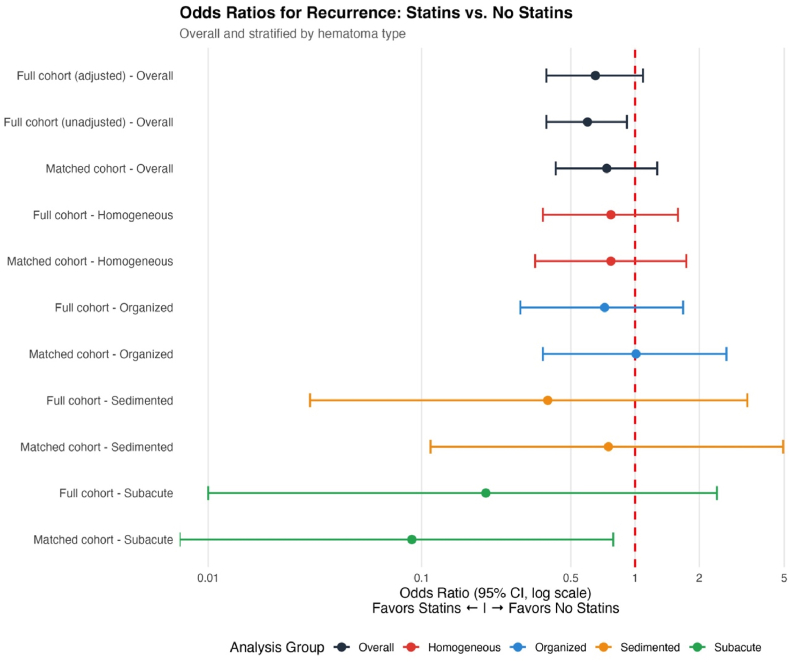
**Forest Plot of Odds Ratios for Recurrence Associated with Statin Use** Odds ratios and 95% confidence intervals for the association between pre-existing statin therapy and postoperative recurrence, displayed on a logarithmic scale. Results shown for overall analyses (black) and stratified by hematoma type: homogeneous (red), organized (blue), sedimented (orange), and subacute (green), comparing full cohort (unadjusted and adjusted for demographics, comorbidities, and hematoma characteristics) and propensity score-matched cohort. The vertical dashed line represents OR = 1.0 (null effect). Point estimates left of the line favor statin use (reduced recurrence). Confidence intervals cross unity in all analyses except matched subacute hematomas (OR 0.09, 95% CI 0.00-0.79, p = 0.052), which approached significance based on 5 events. Despite consistent directional trends favoring statin use, no statistically significant associations were observed after adjustment for confounders and hematoma architecture. Wide confidence intervals in sedimented and subacute subgroups reflect limited sample sizes.

Stratified analyses within the matched cohort revealed consistent patterns with the primary analysis (Fig. 3). Among homogeneous hematomas (n = 95), recurrence rates were 44.4% without statins versus 38.0% with statins (OR 0.77, 95% CI 0.34–1.74, p = 0.524). Organized hematomas (n = 107) showed nearly identical recurrence rates between groups (18.6% vs 18.8%, OR 1.01, 95% CI 0.37–2.68, p = 0.989).

## 4 Discussion

In this retrospective cohort of 564 surgically treated patients with cSDH, we found no significant independent association between pre-existing statin therapy and postoperative recurrence after adjustment for clinical confounders and hematoma architecture. While unadjusted analyses suggested a protective effect, this association attenuated when accounting for cardiovascular comorbidities and further adjustment for hematoma imaging characteristics. PSM corroborated these findings. Hematoma architecture emerged as the strongest predictor of recurrence, with organized and subacute hematomas demonstrating reduced recurrence risk compared to homogeneous types, independent of statin exposure. Formal interaction testing revealed no evidence that statin effects varied significantly across hematoma subtypes. These findings suggest that as a pre-existing, non-modifiable factor at the time of surgical presentation, prior statin exposure offers limited prognostic value for predicting recurrence risk. When evaluating the role of statins in cSDH, a critical distinction should be made between three clinically distinct scenarios: pre-existing statin use prior to cSDH development, initiation of statin therapy after surgical treatment to reduce recurrence, and use of statins as primary medical management in patients with mild symptoms, small hematomas, or those too frail for surgery. The majority of evidence supporting statin efficacy in cSDH pertains to the latter two scenarios, predominantly using atorvastatin as the therapeutic intervention. The ATOCH trial provides evidence for atorvastatin as a medical treatment in patients with mild to moderate cSDH (Jiang et al., 2018). In this randomized controlled trial, atorvastatin given daily for eight weeks resulted in greater hematoma volume reduction compared to placebo. Neurological improvement occurred more frequently in atorvastatin-treated patients, and fewer patients required surgical intervention. Building upon these findings, Wang et al. investigated whether adding low-dose dexamethasone potentiates atorvastatin's effects in a phase II trial of patients with mild to moderate cSDH (Wang et al., 2021b). This approach achieved greater hematoma reduction compared to atorvastatin monotherapy, with substantially more patients achieving complete neurological recovery. The largest real-world evidence of atorvastatin use comes from a Chinese perspective multicenter registry study of over two thousand patients (Liu et al., 2025). Using PSM to address treatment selection bias, atorvastatin was associated with significantly improved functional outcomes, reduced mortality, shorter hospital stays, and lower costs. Notably, however, recurrence rates did not differ between groups, and adverse events were comparable. While these real-world data suggest atorvastatin's effectiveness extends beyond controlled trial settings, the observational design introduces unmeasured confounding despite statistical adjustment, and the lack of recurrence benefit highlights that functional improvement and recurrence prevention may not necessarily align.

### 4.1 Contextualizing our findings

Importantly, the therapeutic context of these trials differs fundamentally from our study's focus on pre-existing statin use. The ATOCH and Wang trials enrolled patients suitable for conservative management and excluded those requiring immediate surgery, antiplatelet therapy, or with bleeding/thrombosis history, whereas our cohort represents the broader surgical population including patients with more severe presentations. Furthermore, the observational nature of our study introduces selection bias related to conditioning on having developed symptomatic cSDH: we only observe patients who developed symptomatic cSDH despite pre-existing statin therapy, potentially representing a population in whom statins' preventive effects, if any, were insufficient. Our findings that pre-existing statin use does not significantly reduce post-surgical recurrence do not necessarily contradict the therapeutic efficacy demonstrated in interventional trials. Rather, they suggest that as a non-modifiable factor at the time of surgical presentation, prior statin exposure offers limited prognostic value for predicting recurrence risk. The dominant predictor in our cohort was hematoma architecture, with organized hematomas showing markedly reduced recurrence risk compared to homogeneous types, a relationship independent of statin status. This indicates that the membrane formation and organization process critical to preventing recurrence operate independently of pre-existing statin exposure.

A fundamental limitation in observational studies of pre-existing medication use is that we cannot observe patients in whom statins may have prevented cSDH formation following minor head trauma, rendering these “prevented cases” invisible to clinical observation. However, prospectively studying this hypothesis would require following large well-characterized cohorts with minor head trauma, a methodologically challenging endeavor given the heterogeneity of traumatic mechanisms and the relative rarity of cSDH development. Our findings contrast with those reported by Guidry et al., who similarly examined prior statin use in surgically treated cSDH patients (n = 111) (Guidry et al., 2021). They observed a greater reduction in hematoma size among statin users at median 30-day follow-up imaging. In their multivariable linear regression adjusting for clinical covariates, statin use was associated with an additional 6.72 mm reduction in maximum hematoma thickness compared to non-users. However, this study measured radiographic hematoma size reduction rather than clinical recurrence requiring reoperation. The smaller sample size and a single follow-up time point may have captured early postoperative radiographic evolution rather than clinically meaningful recurrence. Notably, the authors found no significant differences between groups in repeat surgery rates, 90-day readmissions, or functional outcomes, consistent with our findings. Our study involved examining the interaction between statin use and CT-based hematoma architecture. This analysis was driven by the argument that statins might stabilize immature leaky vessel formation in neomembranes. Our findings showed no evidence that statins differentially affect recurrence risk across hematoma subtypes, independent of the underlying architectural pattern.

### 4.2 Limitations

Several limitations warrant consideration. The retrospective single-center design limits generalizability and introduces center-specific practice patterns and potential selection bias inherent to surgical cohorts, though our PSM sensitivity analysis was designed to mitigate measured confounding. Follow-up was missing approximately 10% of patients, contribution to attrition bias. In addition, statin type, dose, duration of pre-admission use, timing relative to surgical presentation and adherence were heterogeneous and not systematically captured, precluding dose-response analyses. While simvastatin and atorvastatin dominated our cohort, existing trial evidence specifically supports atorvastatin; whether different statins confer differential effects in surgically treated patients remains unknown. Our treatment algorithm might differ from other institutions reducing external validity. Although recurrence was clearly defined there remains some subjectivity in the decision to reoperate or not, which cannot be corrected for. Finally, despite PSM achieving balance on measured covariates, residual unmeasured confounding cannot be fully excluded, as is inherent to observational studies. Because our analysis included only patients who developed symptomatic cSDH requiring surgery, we could not account for patients in whom statin use may have prevented cSDH, which may have led to underestimation of any protective association.

## 5 Conclusion

Pre-existing statin therapy does not independently attenuate postoperative recurrence risk in cSDH in this retrospective observational study after accounting for clinical confounders and hematoma architecture. The apparent association in unadjusted analyses was attenuated after adjustment and was no longer independently observed, which may reflect confounding by comorbidity rather than a direct pharmacologic effect. Hematoma architecture, particularly membrane organization, emerged as the key factor associated with recurrence, independent of statin exposure. While randomized trials have established atorvastatin's efficacy as primary conservative treatment, pre-existing statin exposure offers limited prognostic value for surgical candidates. Whether initiating statin therapy post-surgically reduces recurrence remains unanswered and warrants prospective investigation.

## Consent for publication

Due to the retrospective nature of data collection, informed consent was waived by our local ethical review board.

## Availability of supporting data

The raw data of this analysis can be made available by the authors to any qualified researcher upon reasonable request.

## Authors' contributions

This study was designed and conceptualized by HH and MV.

The manuscript was drafted by HH and MV.

Data collection was performed by HH, HR, KFR, and MV.

All authors were involved in the interpretation and analysis of data.

This study was supervision by MV.

The final version of the manuscript was reviewed, corrected and approved by all authors.

## Funding

This project was made possible by the generous funding of the 10.13039/501100001659German Research Foundation (10.13039/501100001659Deutsche Forschungsgemeinschaft, 10.13039/501100001659DFG), through which Michael Veldeman received a Walter Benjamin Scholarship (Grant Number: VE 1274/1-2).

## Competing interests

There are no conflicts of interest to report.

## Abbreviations

AIC: Akaike information criterion
CI: confidence interval
CT: computed tomography
cSDH: chronic subdural hematoma
GCS: Glasgow coma scale
IQR: interquartile range
OR: odds ratio
Q1: first quartile
Q3: third quartile
SD: standard deviation
SMD: standardized mean difference
VIF: variance inflation factor
